# Engineering Hydrogen Transport Networks in Mg-Based Solid-State Hydrogen Storage: From Activated Interfaces to Hierarchical Architectures

**DOI:** 10.3390/molecules31142522

**Published:** 2026-07-20

**Authors:** Chen Chen, Yunxuan Zhou, Liangjuan Gao, Pingkeng Wu, Zhao Ding

**Affiliations:** 1 Department of Mechanics, Jinzhong University, Jinzhong 030606, China; chenchentgzy@163.com; 2 National Engineering Research Center for Magnesium Alloys, College of Materials Science and Engineering, Chongqing University, Chongqing 400044, China; yunxuanzhou@cqu.edu.cn; 3 College of Materials Science and Engineering, Sichuan University, Chengdu 610065, China; lgao87@scu.edu.cn; 4 Department of Chemical Engineering, Illinois Institute of Technology, Chicago, IL 60616, USA; pwu18@hawk.iit.edu

**Keywords:** activated interfaces, alloying, hierarchical architectures, hydrogen transport networks, magnesium-based hydrogen storage, phase-network engineering

## Abstract

Magnesium-based materials remain among the most intensively studied solid-state hydrogen storage systems because they combine high theoretical hydrogen capacity, elemental abundance, and comparatively low cost. Yet their practical performance is still constrained by sluggish sorption kinetics, difficult hydrogen release, surface passivation, and transport instability under repeated cycling. This perspective argues that these long-standing limitations are most coherently understood not as isolated thermodynamic or kinetic problems, but as a multiscale hydrogen transport-network problem. In this view, hydrogen storage performance depends on whether hydrogen can be admitted, transferred, redistributed, and released through a sufficiently continuous and durable sequence of interfaces, phases, defects, and microstructural pathways. The discussion therefore moves from activated interfaces, which govern hydrogen entry, to phase-network engineering, in which alloying reorganizes internal transport connectivity, and then to hierarchical architectures, where porous hosts, scaffolded secondary phases, and multicomponent microstructures amplify transport efficiency across scales. The perspective further emphasizes that these material-internal transport advantages become meaningful only when they remain compatible with heat and mass transfer at the level of a working storage body and device. Possible descriptors, including active-interface density, connected phase fraction, effective diffusion length, pathway tortuosity, apparent network efficiency, and rate retention during cycling, are further discussed to make this framework more operational. On this basis, the article proposes that future progress in Mg-based hydrogen storage will depend less on isolated optimization of additives or descriptors and more on the deliberate design of connected hydrogen transport networks from the atomic and interfacial scales to the system scale.

## 1. Introduction

Hydrogen storage remains one of the most persistent obstacles between the conceptual appeal of hydrogen as a clean energy carrier and its practical deployment in mobile and stationary energy systems. The problem is not merely one of capacity. A useful hydrogen-storage material must combine acceptable gravimetric and volumetric density with safe operating conditions, reversible uptake and release, tolerable thermal management requirements, and sufficient structural stability under repeated use. These requirements are tightly coupled, and progress in one often comes at the expense of another, which is why the storage question has continued to resist simple solutions even as hydrogen production and utilization technologies have advanced steadily over the past two decades [1,2]. Within the broad family of solid-state hydrogen storage materials, magnesium-based systems have retained a privileged position because they combine several advantages that rarely coexist in the same material family. Magnesium is abundant, lightweight, comparatively inexpensive, and capable, in principle, of storing a large amount of hydrogen through the reversible formation of MgH2. That combination explains why Mg-based materials remain central to both foundational and application-oriented research in solid-state hydrogen storage [3,4,5].

Yet the same material family that appears so attractive in principle has also remained difficult to use in practice. The high enthalpy of hydride formation gives MgH_2_ its appealing hydrogen capacity, but it also makes hydrogen release energetically demanding. Sorption kinetics remain sluggish under many conditions of practical interest, surface passivation hampers the first stages of hydrogen uptake, and repeated hydrogenation and dehydrogenation continuously restructure the material body through stress development, local cracking, grain growth, and redistribution of interfaces. As a result, the apparent promise of Mg-based hydrogen storage has often been accompanied by a frustrating pattern: major improvements can be demonstrated in one descriptor or under one set of conditions, yet the total material response remains difficult to optimize in a coherent and transferable way [3,4,5]. One reason for this difficulty is that the field has often treated key interventions, such as catalysts, alloying additions, nanostructuring, and host-phase engineering, as though they were separate optimization routes whose effects could be interpreted independently. That practice has yielded a large and valuable body of knowledge, but it also tends to obscure the deeper commonality among these strategies.

The motivation for this reinterpretation is not to downplay thermodynamic or kinetic challenges, but to explain why improvements in isolated descriptors often do not translate directly into robust material-level or device-level performance. A lower apparent activation energy, a reduced onset temperature, or a higher density of catalytic sites can be beneficial, but these improvements become practically meaningful only when hydrogen can continuously pass through surface, interfacial, phase, microstructural, and storage-body-scale pathways. The transport-network view therefore reorganizes thermodynamics and kinetics within a multiscale connectivity framework rather than treating them as independent limitations. Compared with catalyst-centered, alloying-centered, nanostructuring-centered, or nanoconfinement-centered approaches, the present framework does not treat each strategy as an independent route for improving one performance descriptor. Instead, it evaluates whether these strategies create connected hydrogen-active nodes and transport links that remain accessible and durable across scales. The key distinction is therefore the shift from optimizing individual additives, phases, or morphologies to assessing network connectivity, bottleneck transfer, and scale translation.

The commonality is transport. In a working Mg-based material, hydrogen does not simply “store” in an abstract host lattice. It must first dissociate at a surface, then cross an interfacial barrier, then move through evolving phases and defects, then redistribute through a microstructure whose geometry and chemistry are changing in real time, and finally be released again through the same or a related pathway. A catalyst, an alloying element, a porous scaffold, a deformation route, or a nanostructure may each improve this overall response, but the improvement is meaningful only if it helps create a more continuous and more accessible route for hydrogen movement. Once viewed in this way, Mg-based hydrogen storage performance becomes less a matter of isolated descriptors and more a matter of network integrity. The central issue is not whether one local step can be accelerated in isolation, but whether hydrogen can move through a sufficiently connected sequence of entry, transfer, redistribution, and release events to yield a practically useful material response [6,7].

This perspective is useful because it provides a common language for strategies that are otherwise easy to discuss in isolation. In this Perspective, a hydrogen transport network is defined as the connected set of hydrogen-active nodes and transport links through which hydrogen is dissociated, admitted, redistributed, accommodated in different phases, and released during cycling. The nodes include surface catalytic sites, Mg/MgH2 and secondary-phase interfaces, defects, grain boundaries, phase boundaries, and porous or scaffolded regions. The links include interfacial transfer routes, bulk or defect-mediated diffusion paths, phase-transformation pathways, and, at larger scale, coupled heat- and mass-transfer routes within the storage body. Catalytic additives matter because they modify entry nodes and interfacial transfer barriers. Nanostructuring matters because it changes diffusion distance, interface density, and defect-mediated pathways. Alloying matters because it reorganizes phase relations and therefore changes the routes through which hydrogen can be redistributed. Hierarchical architectures matter because they determine whether these local advantages remain spatially connected and stable as part of a working material body.

Although such a network cannot usually be reduced to a single descriptor, it can be evaluated semi-quantitatively using several measurable parameters, including active-interface density, effective diffusion length, connected phase fraction, pathway tortuosity, effective hydrogen diffusivity, characteristic sorption time, and retention of kinetic performance after cycling. When a suitable reference state is available, an apparent network efficiency can be expressed as a normalized kinetic or utilization indicator, for example *η*_net_ = *k*_eff_/*k*_ref_ or *U*_bed_ = *C*_bed_/*C*_powder_, under defined and comparable testing conditions. Here, *k*_eff_ represents the experimentally accessible sorption rate constant or characteristic rate of the integrated material or storage body, *k*_ref_ is the corresponding reference value measured for a well-defined baseline material under the same temperature, hydrogen pressure, particle-size range, catalyst loading, and cycling protocol, *C*_bed_ is the usable reversible capacity in the assembled storage body, and *C*_powder_ is the reversible capacity of the corresponding powder or unconstrained material measured under comparable conditions. These ratios should be regarded as operational indicators rather than intrinsic material constants.

In a simplified resistance-type description, the apparent hydrogen flux or overall transport rate may be viewed as being controlled by an effective transport resistance:

*J*_H_ ∝ Δ*μ*_H_/*R*_eff_

*R*_eff_ = *R*_entry_ + *R*_int_ + *R*_phase_ + *R*_micro_ + *R*_bed_

where Δ*μ*_H_ is the hydrogen chemical-potential driving force, *R*_entry_ represents the resistance associated with surface entry and hydrogen admission, *R*_int_ represents interfacial transfer resistance, *R*_phase_ represents phase-to-phase redistribution resistance, *R*_micro_ represents microstructural transport resistance, and *R*_bed_ represents storage-body-scale heat and mass transfer resistance. This expression is not intended as a universal kinetic model. Instead, it provides a conceptual way to show why reducing one local barrier is insufficient if other parts of the multiscale transport pathway remain poorly connected. When these contributions are considered together, the relevant design problem is not simply kinetic enhancement, thermodynamic destabilization, or structural refinement, but the deliberate construction of a hydrogen transport network spanning multiple coupled length scales.

**Figure 1:**
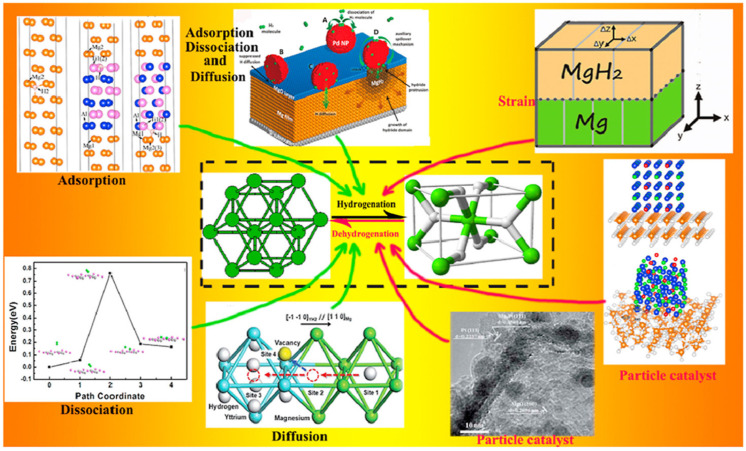
Representative strengthening mechanisms and transport-relevant factors in Mg-based hydrogen storage materials. Adapted from Ref. [6].

That argument is summarized conceptually in Figure 1, which condenses the key transport-relevant strengthening mechanisms discussed in Mg-based hydrogen storage into one integrated framework. The value of such a framework is not that it replaces detailed mechanistic analysis. Rather, it reveals why mechanistically distinct strategies continue to converge on the same practical objective. First-principles analyses have been especially useful in making this convergence visible, because they show that changes in electronic structure, defect state, and local coordination environment eventually manifest as changes in hydrogen adsorption, dissociation, diffusion, and phase stability [6]. In this sense, Figure 1 should not be read as a broad review schematic. It should be read as a transport map. It tells us that the useful unit of analysis is not the additive or the alloying element alone, but the set of connected pathways through which hydrogen can actually move.

Therefore, transport-network connectivity is not proposed as the sole determinant under all conditions, but as a unifying and often decisive factor that determines whether local thermodynamic or kinetic improvements can be translated into effective material- and system-level hydrogen storage performance. Representative descriptors and possible characterization or modeling approaches for such networks are summarized in Table 1. Once the problem is framed this way, the next question becomes much sharper. If Mg-based hydrogen storage performance depends on the quality of the transport network, where does that network begin? It does not begin in the middle of the bulk lattice, nor in a thermodynamic plateau region abstracted from real materials history. It begins at the surface and at the near-surface interfacial region through which hydrogen must first gain entry. In Mg-based systems, where passivation and sluggish dissociation are persistent obstacles, this entry region is not a secondary kinetic detail. It is the first design problem. The discussion that follows therefore begins with activated interfaces and then proceeds inward, from interfacial entry to phase connectivity, from phase connectivity to hierarchical architectures, and finally from material-internal transport to storage performance at the scale of a functional body and system.

The primary bottleneck in such a network is scale-dependent. In unactivated or weakly modified Mg/MgH_2_ powders, the near-surface entry step is often the first bottleneck because surface passivation, sluggish H_2_ dissociation, and interfacial transfer barriers restrict hydrogen admission. Once this entry barrier is reduced by catalysis or high-energy processing, the limiting step may shift to internal redistribution through Mg/MgH_2_ phase boundaries, defect-mediated pathways, or secondary-phase interfaces. At the storage-body level, heat transfer and gas distribution can become equally important or even dominant, particularly when the material is assembled into a packed or compacted bed. This scale-dependent bottleneck shift is a central reason why Mg-based hydrogen storage is treated here as a multiscale transport-network problem.

## 2. Activated Interfaces: The Entry Points of Hydrogen Transport

Hydrogen transport in Mg-based materials begins at the gas–solid interface, and the importance of that fact can hardly be overstated. Before hydrogen can be redistributed through a particle, a grain network, or a multiphase architecture, it must first be dissociated, transferred, and admitted through a near-surface region that is often the most kinetically unfavorable part of the material. Magnesium is readily oxidized, and Mg-based particles commonly present surface layers or contaminated boundary regions that are far less permeable and reactive toward hydrogen than the bulk hydride or metallic host underneath. Even when the thermodynamic driving force for hydrogen uptake is substantial, these near-surface barriers can dominate the observable response. In practice, many of the early-stage bottlenecks in Mg/MgH_2_ are interfacial rather than bulk-limited, which is why so many successful enhancement strategies, though described differently, ultimately operate by rebuilding the entry conditions of the material [8,9,10,11,12,13,14,15].

The role of interfacial engineering becomes especially clear when one compares the effects of different categories of additives and process routes. Transition-metal additions such as Ti-, Nb-, and Ni-containing species can accelerate sorption not simply because they alter one apparent activation barrier, but because they create interfacial conditions in which molecular hydrogen can be more readily dissociated and transferred into the host [9,10,11,12]. Nanostructuring and nanoconfinement are likewise not valuable only because they reduce a characteristic diffusion length. They also increase the density of accessible contact regions, reduce the extent to which passivating surface layers dominate the total response, and amplify the role of interfaces as active rather than merely bounding features [13,14]. Even kinetic interpretations framed in terms of hydriding/dehydriding mechanism eventually return to the same point: if the initial surface and interfacial steps are not accessible, the deeper transport network remains underutilized no matter how favorable the bulk may be [15].

This is why high-energy processing should be interpreted more carefully than it often is. It is easy to describe a route such as ball milling or plasma milling as a preparation method for particle refinement and mixing, but that description captures only part of its significance. High-energy processing also changes what the surface is. It renews contact, exposes fresh regions, strips away contaminated layers, creates lattice defects close to the surface, and may promote in situ formation of catalytically active boundary phases. In other words, it writes the first nodes of the transport network. The resulting entry region differs from that of a simply blended or passivated Mg-based powder not only in morphology, but in local energetic accessibility. Hydrogen does not see the same material when it first encounters a freshly activated, defect-rich, catalytically decorated interface as it does when it encounters a relatively inert or contaminated surface. This difference is not cosmetic. It sets the boundary conditions for every subsequent stage of transport.

**Figure 2:**
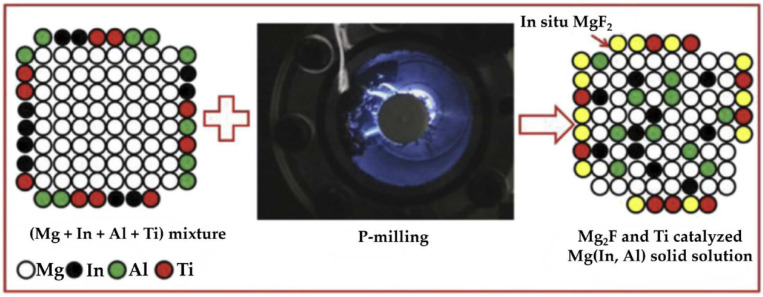
Plasma-assisted milling route and associated interfacial activation in Mg_85_In_5_Al_5_Ti_5_ alloy. Adapted from Ref. [16].

Plasma-assisted ball milling provides an especially clear example because it shows that interfacial activation can be designed deliberately rather than accepted as a side effect of processing. In the Mg_85_In_5_Al_5_Ti_5_ system, the combined action of repeated mechanical impact and plasma discharge simultaneously modifies microstructure, surface condition, and local chemical state [16,17]. The milling route does not simply reduce particle size or homogenize composition. It continuously renews and activates the particle surface, promotes defect generation, perturbs passivating layers, and assists in the formation of highly reactive boundary states. The dual-tuning effect of In, Al, and Ti in this system is therefore inseparable from the route by which the alloy is processed. It is not just the elemental combination that matters; it is the fact that the process itself converts a relatively passive surface into an activated transport entrance. Figure 2 is valuable in this regard because it makes visible the process-to-interface relation that is often hidden beneath shorthand statements about “improved kinetics.” What is being improved is not only a rate constant. It is the physical and chemical accessibility of the first transport step.

Thus, an activated interface should be regarded as the first engineered layer of the hydrogen transport network. Its role is not limited to lowering the H_2_ dissociation barrier; it also disrupts or bypasses passivating surface layers, increases the density of accessible hydrogen-entry sites, promotes spillover- or gateway-type transfer across catalyst/Mg interfaces, and determines whether hydrogen can enter the deeper phase and defect pathways. In this sense, surface activation, process-induced fresh interfaces, and catalyst-decorated nanostructures are useful when they improve both local reactivity and entry continuity.

That interpretation also helps explain why improvements at the interface do not by themselves guarantee high-performance hydrogen storage. An activated surface only solves the first part of the problem. Once hydrogen crosses the boundary, it must still move through the interior of the material. It must encounter a sequence of phases and transformation routes that allow redistribution rather than trapping. It must pass through regions that remain chemically and geometrically compatible with transport. In other words, the network that begins at the surface must continue through the bulk. That continuation is not automatic. It depends strongly on how phase relations are organized, which is why the next level of design must be understood as phase-network engineering.

## 3. Phase-Network Engineering: Alloying as a Route to Connected Transport Pathways

If activated interfaces define where hydrogen first enters, then the next decisive question is what happens after that entry barrier has been crossed. Hydrogen storage in magnesium is often discussed as though the internal response of the material were governed mainly by one bulk phase, with catalysts and additives acting only at the surface or at a few discrete interfacial sites. That view is too narrow for alloyed Mg-based systems. Once alloying is introduced, the material no longer behaves as a single-phase host modified by a small number of local perturbations. It becomes a phase-connected medium in which hydrogen uptake and release are organized through the coexistence, transformation, and interaction of multiple phases. For this reason, alloying should not be interpreted merely as compositional adjustment or as a route to thermodynamic destabilization. Its deeper significance lies in its ability to reorganize internal phase relations and thereby reshape the pathways through which hydrogen is accommodated, transferred, and released. In the present Perspective, this role is described as phase-network engineering.

The value of this reinterpretation is that it shifts the discussion of alloying away from a list of “beneficial elements” and toward a more structural understanding of how hydrogen transport is sustained through the interior of the material. A hydride-forming alloy is not improved only because one phase has a lower enthalpy than another or because one alloying element weakens the Mg–H bond more effectively. It is improved when the phases available during hydrogenation and dehydrogenation form a sequence that hydrogen can traverse without excessive interruption. In a favorable system, hydrogen is not repeatedly trapped at dead ends or forced to cross isolated kinetic bottlenecks between poorly connected regions. Instead, it is redistributed through a linked phase environment in which local thermodynamics, boundary conditions, and transformation routes remain compatible with one another. This is why the most useful alloying strategies in Mg-based hydrogen storage often involve more than a simple shift in one global metric. They reorganize the internal topology of transport.

**Figure 3:**
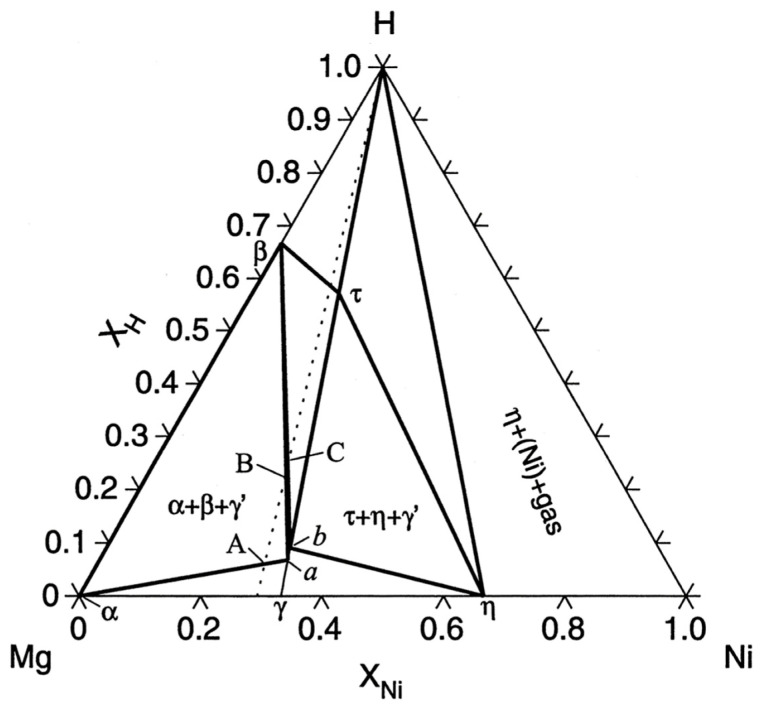
Ternary phase diagram of the Mg–Ni–H system, showing the phase relations associated with hydrogen absorption and desorption in alloyed Mg–Ni materials. Adapted from Ref. [18].

The Mg–Ni–H system remains the clearest illustration of this point. Once nickel is introduced into magnesium, the hydrogen storage problem can no longer be described as a simple extension of pure Mg/MgH_2_ chemistry. The formation of Mg_2_Ni and, under hydrogenation, Mg_2_NiH_4_ creates a distinct phase node with thermodynamic and kinetic characteristics that differ from those of MgH_2_ alone [18,19]. The importance of the Mg–Ni–H ternary phase diagram therefore extends well beyond equilibrium description. As shown in Figure 3, it maps a phase space through which hydrogen absorption and desorption must proceed. The α and β regions, the two-phase coexistence fields, and the absorption/desorption paths shown on the diagram are not merely descriptive labels attached to a ternary system. They define the possible sequences by which hydrogen can move between metallic and hydride-bearing environments. In this sense, the ternary diagram may be read as a transport map as much as a thermodynamic one. It tells us not only which phases are possible, but which phase connections are available for reversible hydrogen exchange.

Once the ternary system is interpreted this way, several features of alloyed Mg-based hydrogen storage become easier to understand. The usefulness of nickel is not exhausted by the statement that Mg_2_NiH_4_ has more favorable desorption behavior than MgH_2_. What matters is that the formation of Mg_2_Ni/Mg_2_NiH_4_ introduces an additional transport-compatible pathway into the system. Hydrogen can now move through a phase network that is structurally and thermodynamically different from the one available in pure magnesium. Similarly, the advantage of alloying with other transition metals should not be judged only by whether they create an intermediate hydride phase or reduce desorption temperature. Their importance depends on whether they reorganize the sequence of accessible states in a way that improves internal continuity. This is why studies on Mg–M systems produced by reactive mechanical alloying often reveal improvements that cannot be understood solely through “catalytic effect” language [20]. The improvement reflects a restructured phase environment rather than only a faster local reaction.

From this perspective, the most promising alloying elements are not necessarily those that optimize one isolated descriptor, but those that create transport-compatible phase relations while preserving capacity and reversibility. Ni remains one of the most representative elements because Mg_2_Ni/Mg_2_NiH_4_ introduces an additional hydride-forming phase node and provides a coupled migration/catalysis pathway. Ti, V, and Nb are also important, particularly when they form catalytically active hydride or interfacial species that facilitate H_2_ dissociation and hydrogen transfer. Rare-earth elements such as Y, Nd, and Ce are attractive because they can form stable hydride-related local environments, refine microstructure, and improve transport durability during cycling. Elements such as Zn, Al, and In may further tune phase constitution and local thermodynamics, although their use must be balanced against capacity loss. Therefore, the most promising direction is likely not a single alloying element, but a coordinated design that combines transition-metal-assisted hydrogen transfer with rare-earth-assisted microstructural stabilization.

This viewpoint also clarifies why combinatorial and composition-guided alloy exploration has remained valuable in Mg-based hydrogen storage research. If hydrogen transport depends on phase connectivity, then composition space is no longer just a list of candidate formulas to be screened empirically. It becomes a design space in which different alloying trajectories create different internal transport landscapes [21]. Some compositions generate favorable intermediate phases but poor connectivity. Others preserve bulk capacity but fail to build an efficient sequence for hydrogen redistribution. Still others may offer an advantageous balance among hydrogen accommodation, transformation enthalpy, and interfacial continuity. In other words, alloying is not just a matter of finding the “best element.” It is a matter of engineering phase architecture.

This phase-architecture perspective also helps explain why alloying effects often appear highly system-specific. The same alloying element may behave very differently depending on whether it enters solid solution, promotes an intermetallic phase, stabilizes a ternary hydride, alters grain morphology, or changes the transport role of pre-existing interfaces. This is one reason that direct comparisons among alloying strategies can be misleading when they are reduced to one descriptor such as equilibrium pressure or activation energy. The more relevant question is whether the alloyed system offers a better-connected route for hydrogen entry, migration, phase accommodation, and release. In Mg–Ni alloys, for example, the presence of Mg_2_Ni is important not only because it changes hydrogen-storage thermodynamics, but because it creates a phase relationship through which hydrogen migration and catalysis can become more strongly coupled [22]. That coupling is the essence of phase-network engineering.

Thus, interface activation and phase-network engineering are complementary. The former provides accessible entry points, whereas the latter determines whether hydrogen can be redistributed through the interior without being trapped in poorly connected phase regions. However, even favorable phase relations may remain too localized or fragile for whole-particle or bed-scale transport. This motivates the hierarchical architectures discussed below, where porous hosts, scaffold-like secondary phases, and multicomponent microstructures extend phase connectivity across larger length scales. 

## 4. Hierarchical Transport Architectures: From Porous Hosts to Multicomponent Microstructures

If interface activation addresses the entry barrier and phase-network engineering addresses internal phase connectivity, then a third level of design becomes necessary when one asks how hydrogen transport can remain efficient beyond isolated reaction sites. This level is best described as hierarchical transport architecture. The term is used here deliberately. The point is not simply that some Mg-based systems possess complicated morphologies or multiple components. The point is that transport becomes more effective when structure is organized across several scales at once, such that local catalytic activity, short-range transfer, phase continuity, defect-mediated migration, and microstructural accessibility reinforce rather than undermine one another. Once this happens, the material is no longer just a modified hydride powder. It becomes an engineered transport body.

One important route to such organization is the use of porous or scaffolded secondary phases. Their significance is often understated when they are described merely as supports or catalyst carriers. In reality, once a secondary phase provides large accessible surface area, interconnected pore space, and stable dispersion of active species, it begins to reorganize the local environment through which hydrogen must move. Transport in Mg-based materials is rarely limited by one elementary step in isolation. Even when dissociation at an active site is improved, overall response may still be constrained by long migration distances, agglomeration of hydride particles, or poor continuity between active and inactive regions. A porous secondary architecture reduces these mismatches by increasing contact density, stabilizing distributed reactive zones, and shortening the distances over which hydrogen must be transferred. This is why MOF-containing MgH_2_ nanocomposites, including 2D MOF@Pd hybrid nanosheets, MgH_2_–Ni MOF catalytic architectures, and MgH_2_–TM MOF (TM = Fe, Ni) composites, are so instructive [23]. Their benefit lies not only in the chemistry of the active species they provide, but in the fact that they create a more continuous environment for hydrogen transfer.

**Figure 4:**
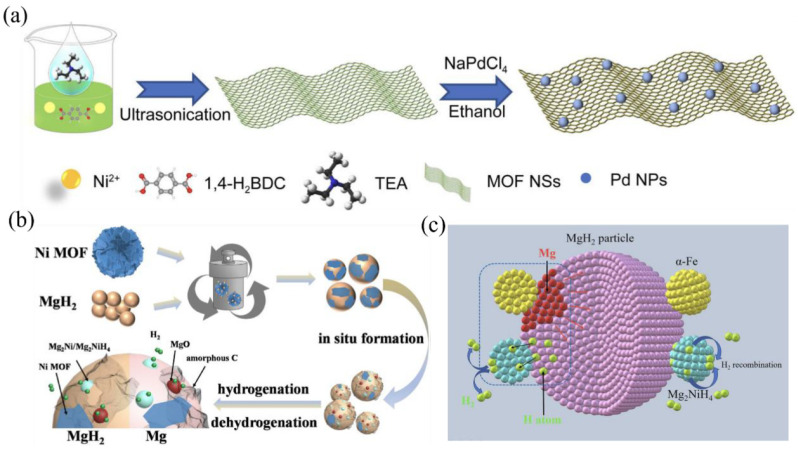
Representative MOF-containing nanocomposite architectures for enhancing hydrogen transport in MgH_2_-based systems. (**a**) Schematic diagram for the preparation of 2D MOF@Pd hybrid nanosheets, highlighting the combination of porous MOF nanosheets and catalytically active Pd nanoparticles. (**b**) Schematic illustration of the catalytic mechanism of MgH_2_–5 wt.% Ni MOF, in which the Ni-MOF-derived architecture provides distributed catalytic contact sites for hydrogen dissociation and transfer. (**c**) Schematic illustration of the catalytic mechanism of MgH_2_–TM MOF (TM = Fe, Ni) composite, where in situ formed active phases and dispersed nanoparticles promote hydrogen dissociation, recombination and migration. Adapted from Ref. [23].

The transport significance of these MOF-containing systems is architectural rather than purely catalytic. A 2D MOF-based heterostructure is useful not simply because it hosts catalytically favorable sites, but because it also defines where those sites are located relative to the MgH_2_ domains that need to exchange hydrogen. Ni-MOF-derived architectures provide distributed catalytic contact states, while MgH_2_–TM MOF (TM = Fe, Ni) composites illustrate how in situ formed active phases and dispersed nanoparticles can promote hydrogen dissociation, recombination, and migration [23]. The consequence is that hydrogen no longer moves only through a conventional compacted powder bed of partly isolated particles. It moves through a scaffolded architecture in which porosity, active interfaces, and catalytic domains are spatially coupled. Figure 4 brings these architectures together not to catalogue MOF-based improvements, but to show that once a secondary phase begins to define access, continuity, and contact density, it becomes part of the transport network itself.

From a practical viewpoint, hierarchical architectures should not be optimized by simply increasing structural complexity. A more realistic strategy is to build the minimum necessary hierarchy using scalable processing routes, such as ball milling, reactive milling, melt spinning, cold rolling, or simple mixing with low-loading secondary phases. Low-cost transition metals, metal oxides, carbonaceous scaffolds, and MOF-derived species are preferable to high noble-metal loadings when similar connectivity and catalytic functions can be achieved. The key variables are catalyst dispersion, pore connectivity, particle size, interfacial contact density, and packing state, which must be optimized together with gravimetric and volumetric capacity. In this sense, hierarchical design should aim at transport effectiveness per unit processing complexity rather than structural sophistication for its own sake.

Porous scaffolds are one route to improved continuity, but they are not the only one. A second and, in some ways, more demanding strategy is to construct a hierarchical microstructure in which transport efficiency is amplified across several structural scales at once. In such systems, hydrogen transport is not improved simply because one interface is more active or one phase is more favorable. It is improved because composition, deformation history, grain structure, secondary-phase distribution, and hydride-forming domains are organized in a way that creates recurrent transport advantages across scales. The result is not only faster local exchange, but a more integrated and more resilient transport body.

Rare-earth-containing magnesium alloys provide some of the clearest examples of this kind of hierarchy. The effect of yttrium content in ball-milled Mg_2.4_–Y_x_Ni alloys cannot be understood solely in terms of one compositional parameter. Changes in Y content alter phase constitution, interfacial character, and hydrogen-storage response in a coupled way [24]. The contrast between as-cast and extruded Mg–Y–Nd–Zr alloys likewise shows that deformation history profoundly changes the resulting microstructure and therefore the conditions under which hydrogen is admitted and redistributed [25]. In Mg–Y–Zn systems, the role of zinc is not exhausted by compositional tuning; it affects phase formation, morphology, and thus the transport pathways available during absorption and desorption [26]. More broadly, optimization of Y and Ni together in magnesium alloys reveals that alloy chemistry becomes especially effective when it contributes not only to local catalytic enhancement, but to a wider reorganization of hydrogen-relevant microstructure [27]. Ce–Mg–Ni systems make the same point from another direction, because the contrast between as-cast and milled alloys shows that the transport response depends strongly on the combined effect of chemistry and structural state rather than on composition alone [28].

**Figure 5:**
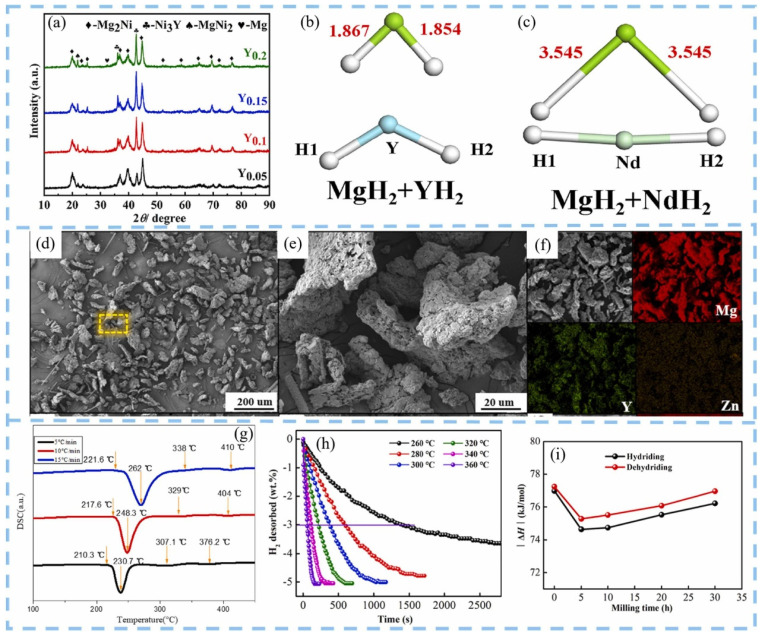
Representative rare-earth-assisted and multicomponent microstructural engineering strategies in Mg-based hydrogen storage alloys. (**a**) XRD patterns showing the phase constitution of ball-milled Mg_2.4_–Y_x_Ni alloys with different Y contents. (**b**) Calculated local H–Y–H configuration in the MgH_2_ + YH_2_ system, illustrating the hydrogen-related local coordination environment introduced by Y-containing hydride species. (**c**) Calculated local H–Nd–H configuration in the MgH_2_ + NdH_2_ system, showing the role of Nd-containing hydride species in modifying the local hydrogen environment. (**d**,**e**) SEM images showing representative microstructural morphologies of rare-earth-containing Mg alloys after different alloying or processing routes. (**f**) Elemental mapping of a Mg–Y–Zn alloy, showing the spatial distribution of Mg, Y, and Zn and the corresponding compositional heterogeneity. (**g**) DSC curves showing the influence of rare-earth-assisted alloying on the thermal dehydrogenation response. (**h**) Isothermal dehydrogenation curves measured at different temperatures, reflecting the kinetic response of the engineered Mg-based alloy. (**i**) Apparent activation energies for hydrogenation and dehydrogenation as a function of milling time, showing the effect of processing history on reversible hydrogen-storage kinetics. Adapted from Refs. [24,25,26,27,28].

What unites these otherwise different cases is that the gain in storage performance is not best interpreted as the sum of isolated effects. It emerges because transport becomes supported at several levels simultaneously. Composition affects which phases can form. Processing affects grain morphology and defect density. Secondary phases influence local catalytic behavior and hydrogen accommodation. Boundaries and interfaces determine how those features connect spatially. Once such a hierarchy is established, hydrogen no longer depends excessively on one isolated local advantage. It moves through a structure in which favorable conditions recur across scales. That is why hierarchical microstructures often outperform systems optimized only at a single level. Their advantage is architectural. This broader point is assembled in Figure 5, where several rare-earth-assisted and multicomponent Mg alloy systems are brought together as examples of microstructural transport engineering. The figure should not be read as a survey of alloy compositions. Its deeper significance is that it shows how phase constitution, morphology, deformation state, and kinetic response become linked when transport is engineered at multiple levels rather than in one isolated domain.

These examples indicate that Mg-based hydrogen storage cannot be optimized by chemistry, defects, or geometry alone. Chemistry defines whether hydrogen can dissociate, transfer, and be accommodated; defects and interfaces provide non-bulk migration routes; and geometry controls whether these favorable features form usable pathways. Their coupling, rather than any single descriptor by itself, determines whether different modification strategies can be compared within the same transport-network framework.

A final point follows directly from this argument. No transport architecture, however sophisticated, is useful unless it remains functionally connected during repeated hydrogenation and dehydrogenation. This requirement is particularly severe for Mg-based systems because cycling continuously perturbs the features that initially provide transport advantage. Volume changes, local stress accumulation, grain growth, redistribution of secondary phases, and gradual loss of active contact may all degrade the continuity of the network [28,29]. Maintaining network stability therefore requires suppressing grain coarsening, catalyst migration, irreversible phase segregation, oxidation, and the loss of interparticle or interphase contact during repeated cycling. For this reason, hierarchical complexity should not be celebrated for its own sake. It has value only when it remains transport-relevant under repeated use. This is the point at which the discussion must move beyond the internal architecture of the material and ask how such architectures translate into usable storage performance at the level of a storage body and, ultimately, a storage unit.

## 5. From Transport Networks to Usable Storage Performance

**Figure 6:**
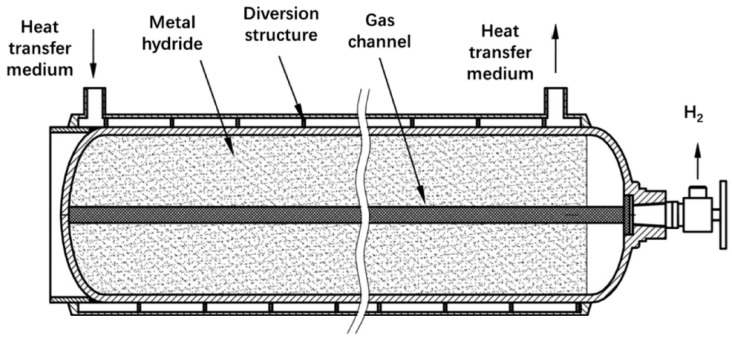
Schematic representation of a solid-state hydrogen storage unit, including the storage vessel, gas-supply line, thermal-management components, and monitoring system. Adapted from Ref. [30].

If the preceding sections establish that Mg-based hydrogen storage performance depends on how effectively hydrogen transport is organized from the surface to the phase-connected and hierarchically structured interior, then a further question becomes unavoidable. What does such transport engineering mean once the material is considered not as an isolated powder or laboratory composite, but as part of a working storage body and, ultimately, a storage unit? This question is not peripheral. It is the point at which many apparently strong laboratory materials begin to diverge from practical usefulness. A Mg-based hydride may show rapid hydrogen uptake in a finely dispersed, catalyst-rich, nanostructured state and yet still perform unsatisfactorily once thermal exchange, gas supply, packing density, geometrical confinement, and repeated operation are considered together. The reason is that useful storage performance is not determined by local reactivity alone. It is determined by whether the transport network written into the material remains compatible with the larger transport environment in which the material must work. As illustrated in Figure 6, a realistic solid-state hydrogen storage unit is not simply a passive container filled with reactive material; it is a coupled system involving gas delivery, heat management, structural containment, and monitoring functions that together determine whether the material’s intrinsic transport advantages can actually be utilized [30].

Seen from this perspective, the importance of transport-network engineering becomes more, not less, pronounced at larger scale. Activated interfaces, favorable phase connectivity, porous scaffolds, and hierarchical microstructures matter because they determine whether hydrogen and heat can continue to move through the material body under realistic conditions. Once the reactive medium is assembled into a finite volume, the meaning of “good kinetics” changes. A locally active catalyst distribution may be insufficient if heat removal is poor. A highly nanostructured powder may not remain transport-efficient if packing state compromises contact continuity. A multiphase architecture may appear advantageous in a thin laboratory specimen but prove less useful if its channels are not preserved in a thicker or more constrained body. This is why the translation from material design to usable storage performance should not be described as a separate engineering layer added after the chemistry is solved. It is the continuation of the same transport problem at a larger scale. Reviews of metal hydrides in fuel-cell-related systems and broader storage applications have repeatedly emphasized that system performance depends not only on reversible capacity and equilibrium thermodynamics, but also on the practical coupling among gas flow, heat exchange, and transport through the hydride bed itself [31,32,33].

This observation also changes how performance descriptors should be interpreted. Gravimetric capacity, onset temperature, apparent activation energy, and local rate constants remain useful, but none of them alone defines whether a Mg-based material will function effectively as a storage body. Once heat must be moved through the body, hydrogen must be delivered through non-ideal pathways, and cycling perturbs contact and microstructure, the relevant question becomes whether the material architecture can preserve usable transport under realistic thermal and geometrical conditions. From this standpoint, a moderately active but structurally robust architecture may outperform a highly active but fragile one when gas access, heat flow, packing state, and cycling durability are considered together.

Several boundary conditions should also be noted. The transport-network view does not imply that transport continuity is always the dominant limitation in Mg-based hydrogen storage. In systems where MgH_2_ remains thermodynamically too stable, the equilibrium dehydrogenation temperature and plateau pressure may still be governed primarily by hydride thermodynamics. In a storage body with insufficient heat exchange, the apparent sorption rate may be controlled more strongly by thermal management than by local hydrogen diffusion. Similarly, excessive catalyst loading, over-refined nanostructuring, or highly porous architectures may improve local transport but reduce practical gravimetric or volumetric capacity. Irreversible oxidation, phase segregation, sintering, or loss of contact during cycling may also degrade performance in ways that cannot be captured by transport continuity alone. Therefore, the hydrogen transport network should be regarded as a design framework that complements, rather than replaces, thermodynamics, capacity, heat management, and cycling durability.

## 6. Outlook: Toward Deliberate Multiscale Transport-Network Design

The broader implication of this Perspective is that Mg-based hydrogen storage should increasingly be interpreted through the continuity, accessibility, and durability of hydrogen transport rather than through isolated improvements of individual descriptors. Catalysts, alloying additions, porous hosts, secondary phases, deformation routes, and rare-earth-assisted microstructures all remain important, but their significance is more coherent when they are understood as ways of building or preserving a connected transport architecture. Activated interfaces matter because they define where hydrogen first enters. Phase-network engineering matters because it determines how hydrogen can be redistributed through the evolving material. Hierarchical architectures matter because they amplify transport efficiency beyond one local catalytic site. System translation matters because a transport network is meaningful only if it remains useful at the scale of a real storage body. When these layers are considered together, the future of Mg-based hydrogen storage appears less like a search for one ideal composition and more like a problem of deliberate multiscale transport design.

This shift in emphasis also suggests more concrete directions for future work. First, hydrogen transport networks should be characterized more directly rather than inferred only from final sorption curves. Operando or in situ XRD and synchrotron-based methods can track the sequence and spatial distribution of phase transformations during hydrogenation and dehydrogenation. Electron microscopy, FIB-SEM tomography, and three-dimensional image reconstruction can determine whether catalytic phases, pores, Mg/MgH_2_ domains, and secondary phases form connected pathways. Isotope-labeling experiments, H/D exchange, neutron-based methods, ToF-SIMS, and related techniques may further clarify where hydrogen enters, migrates, accumulates, or becomes blocked. These approaches are important because they can distinguish a genuinely connected transport architecture from a material that only contains many isolated active sites.

Second, because this Perspective aims to establish a conceptual framework rather than report new simulation data, theoretical simulations are discussed here as validation and quantification tools for the proposed transport-network descriptors. Computational methods should therefore be used to quantify network connectivity across scales. DFT remains useful for evaluating H_2_ dissociation, H adsorption, H migration barriers, defect formation energies, and phase-boundary energetics. Molecular dynamics and kinetic Monte Carlo simulations can describe local diffusion and hopping processes. Phase-field modeling can follow moving Mg/MgH_2_ phase boundaries and stress-assisted microstructural evolution during cycling. Graph-based analysis of reconstructed microstructures could further convert interfaces, pores, defects, and phase domains into nodes and edges, allowing descriptors such as connectivity, tortuosity, bottleneck density, and percolation continuity to be compared among different material designs. At the storage-body level, finite-element models are needed to couple hydrogen transport with heat transfer and gas flow.

Third, new materials should be benchmarked using descriptors that distinguish intrinsic material kinetics from storage-body-level usability. In addition to capacity, onset temperature, plateau pressure, and apparent activation energy, future reports should include active-interface density, connected phase fraction, effective diffusion length, apparent network efficiency, rate retention after cycling, and bed-level utilization when possible. Comparisons should also be made under consistent catalyst loading, particle size, temperature, hydrogen pressure, and cycling protocols. In this way, catalysts, alloys, scaffold-assisted architectures, and rare-earth-enabled microstructures can be evaluated not only by whether they improve one measured number, but by whether they create a more continuous, durable, and transferable route for hydrogen movement across scales.

## Figures and Tables

**Table 1:** Representative descriptors and methods for evaluating hydrogen transport networks in Mg-based hydrogen storage materials.

Network Level	Main Transport Role	Possible Descriptors	Characterization or Modeling Approaches
Activated interface	H_2_ dissociation and hydrogen entry	Active-site density; entry barrier; H adsorption/dissociation energy; surface coverage of catalytic sites	XPS; EPR; TEM/STEM; kinetic fitting; DFT
Phase network	Internal redistribution between metallic and hydride-bearing phases	Connected phase fraction; phase-boundary density; percolation continuity; phase-transformation sequence	XRD/Rietveld analysis; SEM/TEM mapping; tomography; phase-field modeling
Defect and interfacial pathways	Short-range migration and non-bulk transport	Defect density; effective hydrogen diffusivity; migration barrier; interfacial transfer resistance	EPR; positron annihilation; isotope exchange; QENS; DFT/MD
Hierarchical architecture	Multiscale accessibility and shortened transport distance	Pore connectivity; tortuosity; effective diffusion length; dispersion of active domains	BET; FIB-SEM tomography; 3D image reconstruction; image-based simulation
Storage body/system scale	Usable transport under operation	Apparent network efficiency; bed utilization; heat-transfer coefficient; rate retention during cycling	PCT cycling; calorimetry; thermal imaging; finite-element modeling

## Data Availability

No new data were created or analyzed in this study. Data sharing is not applicable to this article.
